# Notes and News

**Published:** 1889-11-09

**Authors:** 


### Notes and News.

Collected and Communicated.

The *New Review* for this month contains an article of Pasteur's on "Rabies." *Blackwood's* contains some amusing pages, "The Diary of an Idle Doctor." The *National Review* contains an article by Mr. Charles Edwardes on "The Lepers of Crete." The *Gentleman's Magazine* gives Mr. Shaw's views on "Teaching the Deaf and Dumb to Talk." The *Contemporary Review* contains a letter from Sir Morell Mackenzie; *Temple Bar* an article on "Centenarianism."

The nature of charity fêtes in Paris has lately been exposed by the caustic pen of M. Aurelien Scholl. Every disaster, he remarks, has its counterpart in entertainments. An explosion of firedamp causes the death of 500 men, and enables 3,000 persons of both sexes to meet and dance. These fêtes, however, bring in but little. The receipts are say, 100,000f. General expenses come up to 80,000, and there are 15,000f. for advertising, and 4,000f. for carriages for the members of the committee. When accounts are settled, about 30f. are left for the victims. This description would also fit some English charity fairs.

The annual meeting of St. Paul's Eye and Ear Hospital, Liverpool, was held on October 29th. The report of the committee, which was read by Mr. P. B. M'Quie, hon. secretary and treasurer, stated that the total receipts for the year amounted to £976 9s. 3d., an increase over last year of £22 14s. 7d. This was, however, more than due to the receipts from patients, which had increased during the year by £38 15s. 8d. The expenditure exceeded the income by £23 8s., and there was a total deficit of £351 0s. 4d., made up of accumulated deficiencies from past years. The summary of cases showed that the total number treated during the past year was 4,162, against 3,973 in the year preceding. The number of attendances by patients was 19,803.

The annual meeting of the Birmingham Orthopœdic and Spinal Hospital was held on October 29th. The income is less than the expenditure, and more subscribers are wanted. The medical report, which was read by Mr. Augustus Clay, stated that the hospital for its size was one of the most complete and perfect of its kind. Notwithstanding the closing of the hospital during the outbreak of scarlet fever, and the grave difficulty of carrying on the work of the institute during the alterations, 1,380 new cases had been admitted during the year, and the attendances had been 5,133, as against 1,366 and 5,021 respectively in the previous year. The number of in-patients and renewals had been 636, a diminution of 36 as compared with 1888, but 25 above the average for the last eight years. The operations totalled 277, an increase of 30.

An interesting ceremony marked the conclusion of the Health Exhibition at Worcester. A clock was presented to Mr. Box, the popular curator. It bore the following inscription: "Sanitary Congress, Worcester, 1889. Presented to Mr. E. L. Box, by a few of the exhibitors, in recognition of his great courtesy and kindness in the discharge of his duties as curator."

There have been some disparaging remarks made lately about the Bradford Workhouse, especially about the female sick wards. The *Bradford Observer* sent a reporter to inspect the buildings. The women's infirmary was erected in 1864, and occupies a separate block, as, indeed, do the male infirmary, the male and female imbecile wards, etc., which are placed parallel with each other. The women's infirmary is of three storeys, lighted from both sides, yet the structure is not altogether convenient. The food is cut up in a draughty passage, and must be cold before the inmates get it. Such slight faults could surely be easily remedied, and the union infirmary be so placed above disparaging remarks.

The Lord Mayor attended in state last week a public meeting of those interested in the Metropolitan Provident Medical Association. Amongst those present were Mr. Montagu, M.P., and the Rev. S. A. Barnett. There are already 14 of these dispensaries situated in various parts of London, and it is proposed to open new branches in the Whitechapel-road and Bethnal Green. Briefly stated, the object aimed at is to provide the members with medical attendance and medicine in return for a small monthly subscription. The drugs supplied are of the best quality, and the dispensaries are in charge of fully qualified medical officers. It is stated that 30,000 patients have already availed themselves of the dispensaries belonging to the association.

The examination for the gold medal presented to the Royal College of Surgeons, Edinburgh, by Colonel William Lorimer Bathgate, in memory of his late father, William McPhune Bathgate, Esq., F.R.C.S.E., known as the "Bathgate Memorial Prize," took place on the 26th ult. The examination was a special written one in *materia medica* and therapeutics, and was open to all candidates who have passed the second professional examination for the triple qualification of the Royal Colleges of Physicians and Surgeons of Edinburgh and Faculty of Physicians and Surgeons of Glasgow during the current year, and who have attended the course of *materia medica* required by the said board at the Edinburgh School of Medicine. Three candidates underwent examination, and the medal has been awarded to Mr. Charles Edward Lester, student of medicine, 14, Melville-terrace, Edinburgh. Through the kindness of Colonel Bathgate this prize will be awarded annually.

How quaint and pretty are the names and titles chosen by our ingenious cousins across the Atlantic! We have just received an American journal published in the interest of charities. It is called *The Sheltering Arms*, and amongst the institutions it mentions in its pages are those named "The Children's Fold," a hospital for children in Eighth-avenue; "The Riverside Rest," a shelter for destitute women; "The Fresh-Air Fund," for boarding out; and "St. John's Guild Floating Hospital." This last really deserves more than a passing mention; it is a large barge moored in New York Bay, and mothers and babies are given an excursion and a rest at this floating hospital throughout the summer. During the season just passed St. John's Guild gave 33 free excursions in its floating hospital, carrying in the aggregate 30,000 sick mothers and children. Not only beautifully named, then, but also beautifully done.

The following vacancies are announced this week, the amount of the salary in each case being given after the name of the institution:

**Resident Medical Officers.**

City of London Hospital, Victoria Park. Medical Officer—£100, board, &c.
City of London Hospital, Victoria Park. House Physician—Board, &c.
Leicester Infirmary. Assistant House Surgeon—£80, board, &c.
Central London Sick Asylum. Assistant Medical Officer—£100, board, &c.
Perth District Asylum. Assistant Medical Officer—£100, board, &c.
Barnstaple Infirmary. House Surgeon—£100, board, &c.

**Non-Resident Medical Officers.**

Gateshead Dispensary. Assistant Surgeon—£120.
Borough of Huddersfield. Medical Officer of Health—£400.
London Hospital, Whitechapel Surgical Registrar—£100.

Dr. Brudenell Carter is said to have prepared his scheme for a small curative hospital for insane patients. The scheme will be looked for with interest, for it is undoubtedly time greater attention was paid to curing instead of confining lunatics.

On November 1st the annual meeting of subscribers to Boston Hospital was held, and the report and statement of accounts were approved. A letter was read from Mr. H. J. Atkinson, M.P., offering to give £60 towards the £200 debt on the hospital if the £140 could be raised. Unluckily, the townspeople have been so frequently solicited to aid this charity, the committee did not feel justified in starting a personal canvass, but before the meeting closed the Mayor and several other gentlemen gave £5 towards clearing the debt.

A special appeal is made for funds for the Hospital for Women, Shaw-street, Liverpool. Three years ago, when the present committee took office, they had a small balance, but, with a subscription list quite inadequate for the ordinary expenses, they have been obliged to draw upon that reserve, and it is now exhausted. They state, from personal knowledge, that the hospital has been managed with the greatest economy. The patients themselves have subscribed largely to the hospital, and it is only now the committee is in dire distress that it asks the ladies of Liverpool to subscribe £200 a year.

The Bishop of Worcester on October 23rd consecrated the new chapel which has been erected in connection with the Droitwich Workhouse. Hitherto the religious services for the inmates have been held in the dining-hall, but this gave rise to many objections, and for some years the erection of a chapel has been anxiously looked for by many. The scheme commenced to take tangible form when the new chaplain (the Rev. E. Lewis) was appointed about two years ago. The guardians looked most favourably upon the scheme. New lavatories have also been built, which will conduce to sanitary improvements.

Taunton and Somerset Hospital has got its nursing institute and casualty ward, but the ever-energetic Dr. Liddon has brought forward new wants. At the late meeting he said that a sort of quarantine ward was wanted for the nurses when they returned from visiting infectious places out of doors, and such a ward would probably have to contain two or three rooms. Then a ward was required for the performance of operations of a certain class, while another special ward was wanted for patients who had got mental disturbances of one sort or another. When a patient was affected with delirium tremens or any similar sort of affection he might seriously upset the other patients in an ordinary ward. Luckily, people are liberal at Taunton, and proud of their hospital.

The annual report of Stockton Hospital for the year ending June 30th, 1889, tells of 473 in-patients and 1,265 out-patients treated. This is an increase of 68 patients since last year. A very interesting sheet in the report shows the cost per patient per day from July, 1881, to the present time. The strange yet satisfactory point shown in this sheet is that the expenses have decreased, though the patients have increased.

According to the *British and Colonial Druggist*, a pernicious system has been introduced into the external department of the Dublin hospitals. Poor people are supplied with their physic free of charge from the hospital dispensary, but respectable folk are charged a fee of sixpence each. The result is that the better-class public imagine that the sixpence actually covers the expense of their medicine and attendance, and have no shame-facedness in accepting public charity, thus causing a substantial loss to the Dublin pharmacies, as all of these people are well able to pay for their doctor.

The inhabitants of Tamworth think that a penny a week should not only pay all their medical fees, but give them a right to insult the doctors of the town. At a recent meeting of the friendly societies of Tamworth, the delegate on the Dispensary Committee said that the new proposals of the dispensary doctors to increase the fees charged to the members were thought to be unfair and unjust, and it was decided to hold that conference in order that the opinion of the whole of the friendly societies should be taken on the matter. Bro. Payne said if the doctors wanted more than a penny a week per member, the clubs would have doctors of their own. Bro. Simpson failed to see that the members of the friendly societies were the recipients of charity. He thought if they refused to agree to the new rules that the doctors would come round. Bro. J. H. S. Hatton was of opinion that the doctors had no right to interfere with the management of the dispensary. So long as the doctors were the servants of the institution, and had a voice on the committee of management, the friendly societies would always be kept in the background. It appeared to him that it was the doctors who wanted to live on the charity of the working classes. Of course the medical men of Tamworth will know how to uphold the dignity of their class in dealing with these members of the existing dispensary.

Lady Violet Greville has been appealing for presents and donations for the Maternity Home, Glanis-road, Shadwell. The description given of the home sounds very cosy and comfortable: "The husbands are permitted to see their wives every evening after work for a few minutes, and finding them clean, comfortable, cheerful, and contented, are easily led to make comparisons with their own homes, and occasionally to reform their habits of life. The women, each with a little plainly furnished room of her own, lose the unwelcome sensation of being a mere unit in a large hospital, and consider themselves as responsible inmates of a quiet home. This home feeling is especially cultivated by the matron, and good results often follow the return of the women to their dwellings. Nearly 300 women were nursed last year in the very contracted premises of the home, and, to show the nature of the care given, we may state—a fact mentioned approvingly by Dr. Playfair, the eminent accoucheur, at the last annual meeting—that, while the average rate of deaths in large hospitals is one in thirty, of home-nursed ladies one in six, the percentage of deaths in the Mothers' Home during the five years of its existence has been nil. We have now determined to buy a freehold house in the Commercial-road, and for the sale, furnishing, and alterations we require a sum of £2,000, for which we venture to appeal to a kindly public." We are sure this appeal will meet with a prompt reply.

